# Measuring Land Change in Coastal Zone around a Rapidly Urbanized Bay

**DOI:** 10.3390/ijerph15061059

**Published:** 2018-05-23

**Authors:** Faming Huang, Boqiang Huang, Jinliang Huang, Shenghui Li

**Affiliations:** 1 Third Institute of Oceanography, State Oceanic Administration, Xiamen 361001, China; huangfaming@tio.org.cn; 2 Coastal and Ocean Management Institute, Xiamen University, Xiamen 361102, China; bqhuang@stu.xmu.edu.cn (B.H.); lishenghui1227@163.com (S.L.)

**Keywords:** land change, urbanization, intensity analysis, coastal area

## Abstract

Urban development is a major cause for eco-degradation in many coastal regions. Understanding urbanization dynamics and underlying driving factors is crucial for urban planning and management. Land-use dynamic degree indices and intensity analysis were used to measure land changes occurred in 1990, 2002, 2009, and 2017 in the coastal zone around Quanzhou bay, which is a rapidly urbanized bay in Southeast China. The comprehensive land-use dynamic degree and interval level intensity analysis both revealed that land change was accelerating across the three time intervals in a three-kilometer-wide zone along the coastal line (zone A), while land change was fastest during the second time interval 2002–2009 in a separate terrestrial area within coastal zone (zone B). Driven by urbanization, built-up gains and cropland losses were active for all time intervals in both zones. Mudflat losses were active except in the first time interval in zone A due to the intensive sea reclamation. The gain of mangrove was active while the loss of mangrove is dormant for all three intervals in zone A. Transition level analysis further revealed the similarities and differences in processes within patterns of land changes for both zones. The transition from cropland to built-up was systematically targeted and stationary while the transition from woodland to built-up was systematically avoiding transition in both zones. Built-up tended to target aquaculture for the second and third time intervals in zone A but avoid Aquaculture for all intervals in zone B. Land change in zone A was more significant than that in zone B during the second and third time intervals at three-level intensity. The application of intensity analysis can enhance our understanding of the patterns and processes in land changes and suitable land development plans in the Quanzhou bay area. This type of investigation is useful to provide information for developing sound land use policy to achieve urban sustainability in similar coastal areas.

## 1. Introduction

The land change has led to increased coastal vulnerability [1,2,3,4] and degradation of coastal and marine ecosystems such as the loss of coastal wetlands, water quality deterioration, the decline of biodiversity, habitat destruction, etc. Knowledge concerning how underlying processes drive patterns of land changes has become essential to coastal planning and management. Several methods have been applied to evaluate land changes. Annual intensity of urban expansion intensity has frequently been used to measure rates of urban land expansion [5]. Land-use dynamic degree index has also been applied to represent the urban expansion rate [6]. The area-weighted centroids of land use types have been calculated to investigate the temporal changes in the spatial distribution of anthropogenic land cover in low-elevation coastal zones [7]. An urban growth model was adopted to measure urban expansion rate in Jing-Jin-Ji urban agglomeration [8]. Most of these studies focused their objectives on the patterns of land changes and the underlying causes. However, some of their metrics combine various patterns of change into one measurement, which can make interpretation challenging [9,10].

Intensity analysis is a framework that separates measurements of changes according to distinct levels, each with its own distinct interpretation [11]. Intensity analysis has been applied widely to gain insight into systematic processes of land-cover transitions in many countries including Ghana, Australia, Colombia, Japan, and China [9,12,13,14,15,16,17]. Intensity analysis can be useful in assessing the evidence for a particular hypothesized process of change and can help to develop new hypotheses concerning processes of change [18]. Alo and Pontius (2008) [19] used Intensity Analysis to identify systematic land transitions both inside and outside a protected area in Ghana, thereby verifying the hypothesis that logging is the main cause of the loss of closed forest inside the protected areas whereas farming is the main cause of the loss of closed forest outside the protected areas. Huang et al. (2018) [9] also applied this method to measure land change inside and outside the coastal zone of Longhai.

Quanzhou is a coastal city with a glorious past located in Fujian province in Southeast China, near the Taiwan Strait. Historically, the Quanzhou city was the hub of a thriving trade route between China and the rest of the world, known as the starting point of Maritime Silk Road. Quanzhou is rich in natural and cultural resources due to its unique geography and history. The mangrove reserve area on the coast of the Luoyang Jiang River estuary is essential to maintain a balanced coastal ecosystem for Quanzhou bay. Woodland and farmland protection areas near the bay have received great attention. Recent assessments indicated that the ecosystem of coastal area in Quanzhou bay has been seriously deteriorated due to urbanization and reclamation in the past several decades, especially in areas adjacent to the coastline.

The objectives of this study are (1) to link the patterns with processes in land changes in coastal zone around Quanzhou bay; (2) and to examine the underlying driving mechanisms, which can provide spatial insights for land use policy.

## 2. Materials and Methods

### 2.1. Study Area

Rapid urbanization, industrialization, and extensive reclamation have resulted in severe loss of cropland, mudflat, and wetland in the Quanzhou Bay area, one of the fastest economically developing areas in Fujian Province.

**Figure 1:**
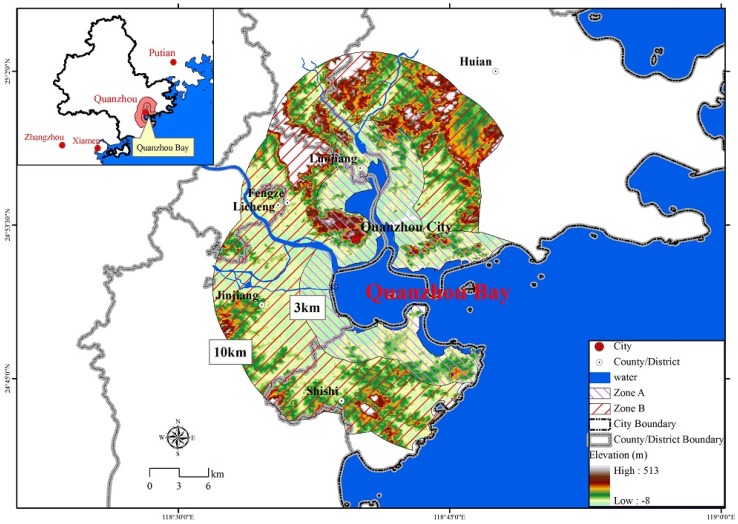
Location of study area.

Figure 1 shows the location of Quanzhou bay and its two zones selected for this study. The boundary of coastal zone terrestrial area in Quanzhou bay is 10 km wide from the coastline extends to the inland area according to the scope of China’s coastal survey [20]. We did a comparison of remote sensing images of Quanzhou bay for the past decades. We found that a three-kilometer-wide area along the coastline has experienced intensive sea reclamation and industrialization. The marine environment and natural coastline have suffered serious damage. Thus, two separate coastal zones are established for this study. The ‘zone A’ is the 179.02 square kilometer area that is 3 km wide along the coastline. The ‘zone B’ is the 437.42 square kilometer area that is the part of Quanzhou bay coastal zone terrestrial area not inside zone A. Both zones experienced acute anthropogenic land changes over recent decades due to rapid economic development.

### 2.2. Data and Pre-Processing

Multi-temporal satellite images on four Landsat Thematic Mapper (TM) or Enhanced Thematic Mapper plus (ETM+) from 1990, 2002, 2009, and 2017 were used to detect land use change in Quanzhou bay coastal area from 1990 to 2017. Table 1 describes the satellite images that serve as the basis for the maps of land categories. These Landsat images were from the Center for Earth Observation and Digital Earth (CEODE), Chinese Academy of Sciences (http://cs.rsgs.ac.cn/cs_cn/), and the United States Geological Survey (USGS) (http://eros.usgs.gov/).

Before land classification was done, the acquired images were processed for atmospheric correction and geo-rectified. All acquired images were geo-rectified with reference to topographic maps, using at least 30 ground control point (GCPs) in each image, such as road intersections and stream confluences. The root mean squared errors of geometric rectification were less than half a pixel as a result of using the first-order polynomial nearest neighbor algorithm with 32 ground control points. All images were re-sampled to a 20 m resolution before the following steps.

A hierarchical classification system was applied to Landsat images to map land category for 2017. Land category in Quanzhou bay area includes woodland, cropland, water, built-up, aquaculture, mudflat, and mangroves. A linear stretching based on spectral characteristics was firstly applied to separate layers into woodland, water, and others. The Iterative Self Organizing Data Analysis Technique Algorithm (ISODATA) was then used to perform unsupervised classification for each layer. This step produced 150 clusters, and then each cluster was assigned into one of seven land categories based on visual interpretation of high-resolution images available on Google Earth.

The generated 2017 map was used to classify each of preceding years in sequence. The map of 2017 was overlaid on the 2009 image and then the visual interpretation was used to classify pixels with the same characteristics. This procedure was repeated to generate land cover maps at 1990, 2002, 2009, and 2017 in sequence. Finally, post-classification comparison was applied to detect categorical transitions by overlaying pairs of land cover maps. All the operations mentioned above were performed using the ERDAS 9.2 Imagine.

### 2.3. Land Use Dynamic Degree Indices

Three indices commonly used were adopted to measure land-use dynamic and urban expansion patterns, including single land use dynamic degree (K) and comprehensive land-use dynamic degree (S) [10,21,22], and annual intensity of urban expansion intensity (SI) [5,23,24,25]. Table 2 presents the notation and equations for these indices.

### 2.4. Intensity Analysis

The intensity analysis method was applied to characterize the observed patterns during the quantitative phase of research and influences all subsequent steps. Below are the equations for intensity analysis, which is a hierarchical framework combining three levels of analysis [11]. The interval level examines how the speed of change, *S_t_*, during each time interval compares to a uniform speed of change, *U*, during the entire temporal extent. During each time interval, *t*, the category level examines how the gross loss intensity, *L_ti_*, from category *i* and the gross gain intensity, *G_tj_*, to category *j* compares to a uniform intensity, *S_t_*. During each time interval, *t*, the transition level examines how the transition intensity, *R_tin_*, from category *i* to category *n* compares to a uniform transition intensity *W_tn_* given the gross gain of category *n*. The transition level also examines how the transition intensity *Q_tmj_* from category *m* to category *j* compares to a uniform transition intensity *V_tm_* given the gross loss of category *m*.

(1) $$U=\frac{\sum_{t=1}^{T-1}\left\{\sum_{j=1}^{J}\left[(\sum_{i=1}^{J}C_{tij})-C_{tjj}\right]\right\}/\left\{\sum_{j=1}^{J}\left(\sum_{i=1}^{J}C_{tij}\right)\right\}}{Y_{T}-Y_{1}}100\%\;=\frac{\mathrm{change}\;\mathrm{area}\;\mathrm{during}\;\mathrm{all}\;\mathrm{intervals}/\mathrm{spatial}\;\mathrm{extent}\;\mathrm{area}}{\mathrm{duration}\;\mathrm{of}\;\mathrm{all}\;\mathrm{intervals}}100\%$$

(2) $$S_{t}=\frac{\left\{\sum_{j=1}^{J}\left[\left(\sum_{i=1}^{J}C_{tij}\right)-C_{tjj}\right]\right\}/\left\{\sum_{j=1}^{J}\left(\sum_{i=1}^{J}C_{tij}\right)\right\}}{Y_{t+1}-Y_{t}}100\%\;=\frac{\mathrm{change}\;\mathrm{area}\;\mathrm{during}\;\mathrm{interval}\;\left[Y_{t,}Y_{t+1}\right]/\mathrm{spatial}\;\mathrm{extent}\;\mathrm{area}}{\mathrm{duration}\;\mathrm{of}\;\mathrm{interval}\left[Y_{t,}Y_{t+1}\right]}100\%$$

(3) $$G_{tj}=\frac{\left[\left(\sum_{i=1}^{J}C_{tij}\right)-C_{tjj}\right]/\left(Y_{t+1}-Y_{t}\right)}{\sum_{i=1}^{J}C_{tij}}100\%\;=\frac{\mathrm{area}\;\mathrm{of}\;\mathrm{gross}\;\mathrm{gain}\;\mathrm{of}\;j\;\mathrm{during}\;\left[Y_{t},Y_{t+1}\right]/\mathrm{duration}\;\mathrm{of}\;\left[Y_{t},Y_{t+1}\right]}{\mathrm{area}\;\mathrm{of}\;j\;\mathrm{at}\;\mathrm{time}\;Y_{t+1}}100\%$$

(4) $$L_{ti}=\frac{\left[\left(\sum_{j=1}^{J}C_{tij}\right)-C_{tii}\right]/\left(Y_{t+1}-Y_{t}\right)}{\sum_{j=1}^{J}C_{tij}}100\%\;=\frac{\mathrm{area}\;\mathrm{of}\;\mathrm{gross}\;\mathrm{loss}\;\mathrm{of}\;i\;\mathrm{during}\;\left[Y_{t},Y_{t+1}\right]/\mathrm{duration}\;\mathrm{of}\;\left[Y_{t},Y_{t+1}\right]}{\mathrm{area}\;\mathrm{of}\;i\;\mathrm{at}\;\mathrm{time}\;Y_{t}}100\%$$

(5) $$W_{tn}=\frac{\left[\left(\sum_{i=1}^{J}C_{tin}\right)-C_{tnn}\right]/\left(Y_{t+1}-Y_{t}\right)}{\sum_{j=1}^{J}\left[\left(\sum_{i=1}^{J}C_{tij}\right)-C_{tnj}\right]}100\%\;=\frac{\mathrm{area}\;\mathrm{of}\;\mathrm{gross}\;\mathrm{gain}\;\mathrm{of}\;n\;\mathrm{during}\;\left[Y_{t},Y_{t+1}\right]/\mathrm{duration}\;\mathrm{of}\;\left[Y_{t},Y_{t+1}\right]}{\mathrm{area}\;\mathrm{of}\;\mathrm{not}\;n\;\mathrm{at}\;\mathrm{time}\;Y_{t}}100\%$$

(6) $$R_{tin}=\frac{C_{tin}/\left(Y_{t+1}-Y_{t}\right)}{\sum_{j=1}^{J}C_{tij}}100\%\;=\frac{\mathrm{area}\;\mathrm{of}\;\mathrm{transtion}\;\mathrm{from}\;i\;\mathrm{to}\;n\;\mathrm{during}\;\left[Y_{t},Y_{t+1}\right]/\mathrm{duration}\;\mathrm{of}\;\left[Y_{t},Y_{t+1}\right]}{\mathrm{area}\;\mathrm{of}\;i\;\mathrm{at}\;\mathrm{time}\;Y_{t}}100\%$$

(7) $$V_{tm}=\frac{\left[\left(\sum_{j=1}^{J}C_{tmj}\right)-C_{tmm}\right]/\left(Y_{t+1}-Y_{t}\right)}{\sum_{i=1}^{J}\left[\left(\sum_{j=1}^{J}C_{tij}\right)-C_{tim}\right]}100\%\;=\frac{\mathrm{area}\;\mathrm{of}\;\mathrm{gross}\;\mathrm{loss}\;\mathrm{of}\;m\;\mathrm{during}\;\left[Y_{t},Y_{t+1}\right]/\mathrm{duration}\;\mathrm{of}\;\left[Y_{t},Y_{t+1}\right]}{\mathrm{area}\;\mathrm{of}\;\mathrm{not}\;m\;\mathrm{at}\;\mathrm{time}\;Y_{t+1}}100\%$$

(8) $$Q_{tmj}=\frac{C_{tmj}/\left(Y_{t+1}-Y_{t}\right)}{\sum_{i=1}^{J}C_{tij}}100\%\;=\frac{\mathrm{area}\;\mathrm{of}\;\mathrm{transtion}\;\mathrm{from}\;m\;\mathrm{to}\;j\;\mathrm{during}\;\left[Y_{t},Y_{t+1}\right]/\mathrm{duration}\;\mathrm{of}\;\left[Y_{t},Y_{t+1}\right]}{\mathrm{area}\;\mathrm{of}\;j\;\mathrm{at}\;\mathrm{time}\;Y_{t+1}}100\%$$

where *J* = number of categories; *i* = index for a category at an initial time; *j* = index for a category at a subsequent time; *m* = index for the losing category in the transition of interest; *n* = index for the gaining category in the transition of interest; *T* = number of time points; *t* = index for a time point, which ranges from 1 to *T*−1; *Y_t_* = year at time point *t*; *C_tij_* = number of pixels that transition from category *i* at time *Y_t_* to category *j* at time *Y_t_*_+ 1_; *S_t_* = annual intensity of change for time interval [*Y_t_*, *Y_t_*_+ 1_]; *U* = value of uniform line for time intensity analysis of [*Y*_1_, *Y_t_*]; *G_tj_* = annual intensity of gross gain of category *j* for time interval [*Y_t_, Y_t_*_+ 1_]; *L_ti_* = annual intensity of gross loss of category *i* for time interval [*Y_t_, Y_t_*_+ 1_]; *R_tin_* = annual intensity of transition from category *i* to category *n* during time interval [*Y_t_*, *Y_t_*_+ 1_] where *i* ≠ *n*; *W_tn_* = value of uniform intensity of transition to category *n* from all non-*n* categories at time *Y_t_* during time interval [*Y_t_*, *Y_t_*_+ 1_]; *Q_tmj_* = annual intensity of transition from category m to category *j* during time interval [*Y_t_, Y_t_*_+ 1_] where *j* ≠ *m*; *V_tm_* = value of uniform intensity of transition from category *m* to all non-*m* categories at time *Y_t_*_+ 1_ during time interval [*Y_t_, Y_t_*_+ 1_].

## 3. Results

### 3.1. Land Use Change Mapping Results

**Figure 2:**
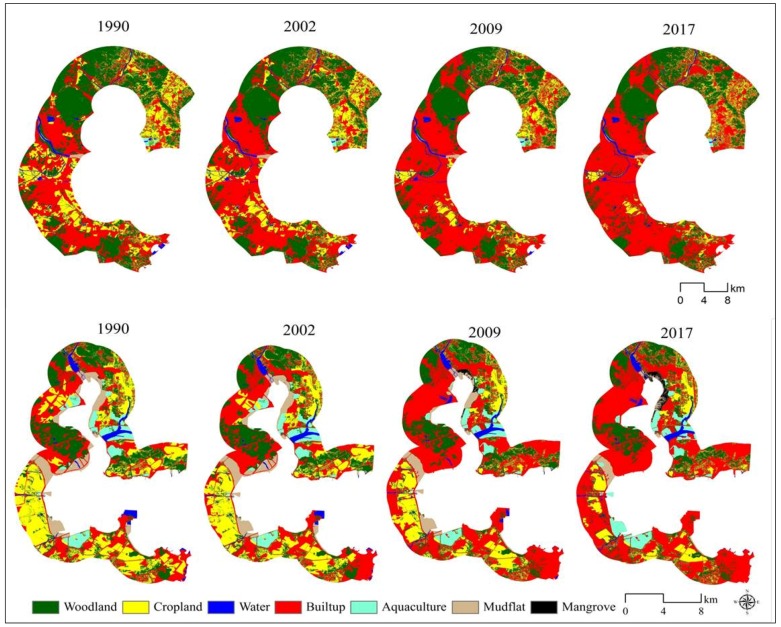
Maps of land categories and changes. Below—the zone A of study area. Above—zone B of the study area.

Figure 2 and Table 3 present maps and percentage of the seven land categories at the four time points of two zones. The accuracy assessment revealed an overall accuracy of 90.12%, 89.69%, 89.46%, and 91.62% for the years 1990, 2002, 2009, and 2017, respectively. This is considered acceptable for this study. As is shown on Figure 2 and Table 3, built-up land was the only land use type that had increases for all three time intervals, and with the highest increase in both two study areas. For zone A, the built-up land increased from 29.2% of the total area to 56.3% from 1990 to 2017. For zone B, the built-up land increased from 38.1% of the total area to 56.0% from 1990 to 2017. This suggests that both zones experienced drastic urban expansion during the study period. Table 3 reveals that woodland and cropland had consistent net decrease in both zones, especially for the cropland. Water body and mudflat had consistent net decrease due to high intensity reclamation, whereas the mangroves had a net increase from 0.01% of the total area to 1.10% during 1990–2017 in zone A.

### 3.2. Results from Land-Use Dynamic Degree Indices

Table 4 lists the value for the land-use dynamic degrees and annual intensity of urban expansion. The comprehensive land use dynamic degree (S) value was the highest at 9.24% in 2002–2009 in zone B, but the value was highest at 15.74% in 2009–2017 in zone A among three time intervals. These results indicate that Quanzhou bay coastal zone experienced intensive land transformation over the last three decades, especially the second period in zone B and the third period in zone A. The land change in zone A was more significant than that in zone B during the second and third intervals (Table 4). In terms of single land-use dynamic degree, woodland, cropland, water, and mudflat, all had negative values while built-up and mangrove had all positive values for both zones in all three time intervals. This may indicate that built-up tends to replace woodland, cropland, water, and mudflat and avoid mangroves induced by urban sprawl. Aquaculture had all negative values except in 2009–2017 in zone A for all three time intervals. Both zones had the highest value of built-up in 2002–2009 among three time intervals, indicating that both zones experienced fastest urban expansion during second periods.

### 3.3. Three-Level Analysis of Land Change Measured by Intensity Analysis

**Figure 3:**
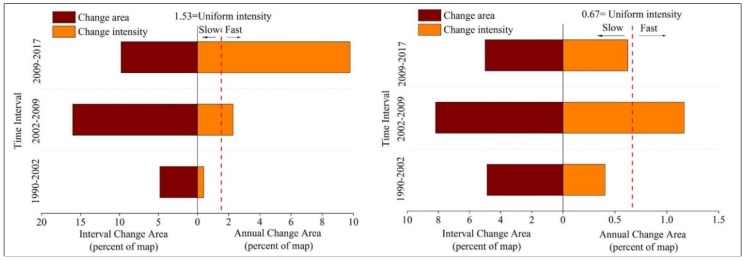
Interval level intensity analysis for three time intervals. **Left**—zone A of coastal zone. **Right**—zone B of coastal zone.

Figure 3 summarizes the results from the interval level intensity analysis. The graph on the left in Figure 3 shows that land change in zone B is the fastest during the second interval 2002–2009, whereas land change is relatively slow for the first and third intervals, especially for the interval 1990–2002. While the graph on right of Figure 3 shows the overall land transformation is accelerating across the three time intervals in zone A. As shown in Figure 3, land change in zone A was more intense than that in zone B during the second and third intervals.

**Figure 4:**
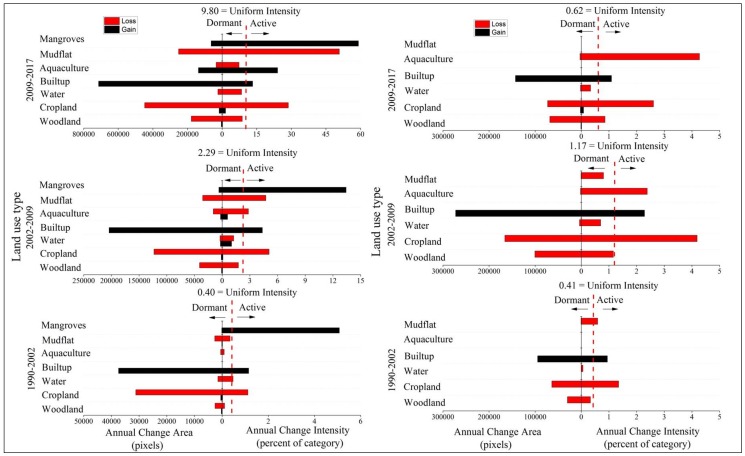
Category level intensity analysis for three time intervals. **Left**—zone A of coastal zone. **Right**—zone B of coastal zone.

Figure 4 shows the results from the category level intensity analysis, which gives one graph per time interval. Graph on the left of Figure 4 shows that built-up gains and cropland losses are active while the gains and losses of woodland is dormant for all time intervals in zone A. This indicates that woodland experienced less intensively gains and losses than if the overall change were to have been distributed uniformly across the landscape. The intensities of mudflat losses are active except the first time interval due to the high-intensity sea reclamation. The gain of mangrove is active while the loss of mangrove is dormant for all three intervals, indicating that mangrove experienced more intensively gains and less intensively losses than if the overall change were to have been distributed uniformly across the landscape. This suggests that mangrove protection programs in this area have been effective during the past three decades. These results are consistent for all three time intervals, meaning that the pattern is stationary at a category level intensity analysis.

Graph on right of Figure 4 shows that cropland and built-up are active for all three time intervals and experience the largest losses and gains in zone B. The intensities of woodland and aquaculture losses are dormant except the third time interval. The intensity of water gains and losses is dormant for all time intervals due to a minor presence of water in both zones. This indicates that water experienced less intensive gains and losses than if the overall change were to have been distributed uniformly across the landscape.

For each time interval, the transition intensity level analysis produced two sets of output. One set analyzes transitions for gains of category *n*, and the other set analyzes transitions for losses of category.

**Figure 5:**
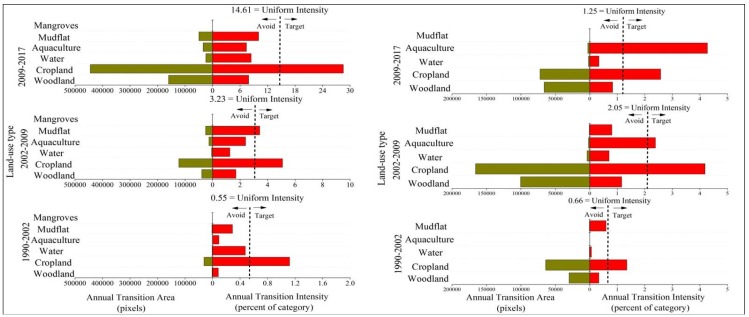
Transition level results for built-up gain. **Left**—zone A of coastal zone. **Right**—zone B of the coastal zone; green bars: annual transition area (pixels); red bars: annual transition intensity (percent of category).

**Figure 6:**
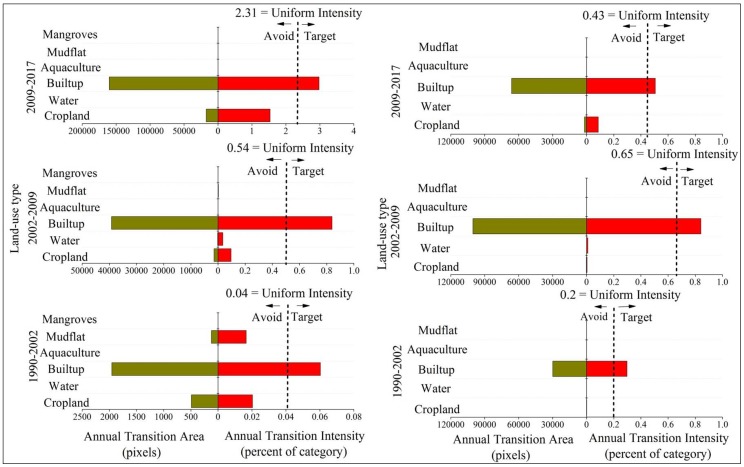
Transition level results given gross woodland loss. **Left**—zone A of the coastal zone. **Right**—zone B of the coastal zone; green bars: annual transition area (pixels); red bars: annual transition intensity (percent of category).

**Figure 7:**
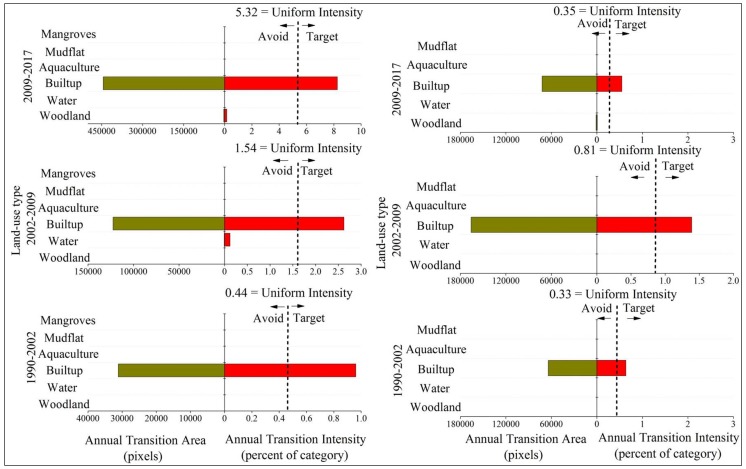
Transition level results given gross cropland loss. **Left**—zone A of coastal zone. **Right**—zone B of the coastal zone; green bars: annual transition area (pixels); red bars: annual transition intensity (percent of category).

**Figure 8:**
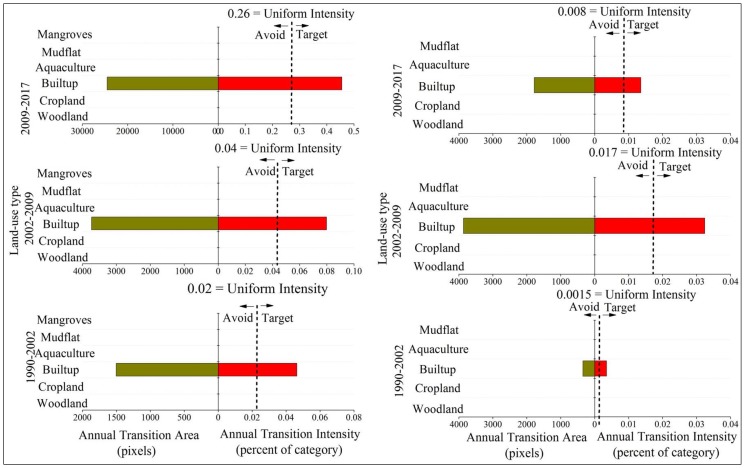
Transition level results given gross water loss. **Left**—zone A of coastal zone. **Right**—zone B of the coastal zone; green bars: annual transition area (pixels); red bars: annual transition intensity (percent of category).

The focus was placed on the transitions from woodland, cropland, mudflat, and water to built-up, as urbanization is intensive in coastal regions throughout the world. Figure 5 shows the analysis of the gains to built-up in three time intervals for both zones within Quanzhou bay coastal areas. The remaining three figures (Figure 6, Figure 7 and Figure 8) represent the analysis of losses from woodland, cropland, and water for the same intervals in both zones. As shown in Figure 5, built-up tends to target cropland more intensively than other categories and avoid woodland for all time intervals when built-up gains in both two zones. Thus, we explain the transition from cropland to built-up as a combination of cropland losing intensively to built-up and built-up gaining intensively from cropland. Therefore, the transition from cropland to built-up is systematic in two zones. In addition, the transition from woodland to built-up is systematically avoiding transition. It should be noted that built-up tends to target aquaculture in the second and latest interval in zone A and tends to target mudflat in the second interval 2002–2009. This indicated specific land transformation processes induced by reclamation in coastal cities. Despite built-up tending to avoid water in zone A, the intensity of water’s losses increased gradually during our study periods. This also suggests an accelerating intensity of reclamation in Quanzhou bay. In general, the transitions from other categories to built-up in zone A are more intensive than transitions in zone B.

## 4. Discussion

### 4.1. Land-Use Dynamic Degree Indices vs. Three-Level Intensity Analysis for Land Change Measurement

Analysis with land-use dynamic indices can provide overall understanding on the land use-change dynamic degree and urban expansion rate and intensity. These indices can support a quantitative assessment of spatial and temporal patterns of land use dynamics over time [6,21,23,24,25]. These indices can also reveal dynamics for a given category in a given time interval, which is basically in agreement with the interval level and category level intensity analysis (Table 4, Figure 3 and Figure 4). The landscape in zone A was underwent more anthropogenic disturbances, the single land-use dynamic degree in terms of woodland, cropland, built-up, mudflat, and mangrove over time were generally larger than the other land types inside zone A of Quanzhou bay coastal area (Table 4). This is similar to the category level intensity analysis that these categories were all active in both zones. Specifically, the comprehensive land-use dynamic degree in 1996–2002 was greatest for zone B while it accelerated in zone A over three time intervals. However, we need more signals to link patterns with processes in land changes.

Intensity analysis revealed the underlying processes of land changes. This method made the comparison of processes driving land changes possible. For both zones, the transition from cropland to built-up is systematic and the transition from woodland to built-up is systematically avoiding transition in all three time intervals; built-up tends to target aquaculture in the second 2002–2009 and latest interval 2002–2010 in zone B while built-up tends to target the loss of mudflat in the second time interval in zone A. In zone A, the transitions from mangrove to built-up are systematically avoiding transitions.

This section discusses the results for land-use dynamic indices and each level of intensity analysis in the context of possible processes. The results for land-use dynamic indices and interval level intensity analysis suggest that the land changed most rapidly during the middle time interval (2002–2009) and latest time interval (2009–2017) for zone A and zone B of Quanzhou bay coastal areas, respectively. Quan et al. (2015) [6] analyzed land change intensity for whole Quanzhou city, and found that annual land change was more intensive during the 2000–2005 time interval. The results of this study in zone B are in agreement with this finding. Huang et al. (2018) [9] analyzed land change in Longhai coastal zone during three time intervals defined by 1986, 1996, 2002, and 2010, and found that land change intensity was greatest during the middle time interval 1996–2002. Nevertheless, current study results show that land change intensity was greatest during 2009–2017 in zone B, which might be due to the fact that Quanzhou coastal area experienced more drastic land change than that for the Longhai coastal area over the past decade.

### 4.2. Driving Factors for Coastal Land Use Intensity Trends and Spatial Patterns

In general, four types of driving factors are considered for driving land change, they are: (1) physical factors, (2) socioeconomic and population growth, (3) surrounding land use type, and (4) land use policy and urban planning [26,27,28]. In this study, elevation, surrounding land use type, urban planning, and land use policy were selected to examine the driving forces leading to the disparities of land changes between two zones.

Physical factors (e.g., climate, elevation, and topography) are the fundamental consideration in the process of spatial expansion of urban land. Previous studies revealed that elevation has significantly negative effects on urban expansion, indicating that the steep and elevated areas are less likely to be developed [29,30]. This negative effect may be due to the cost of development in elevated areas being higher than that in flat areas. In contrast, the effects of elevation on urban expansion depend on the topography of the studied area. There is an obvious distinction between the two zones in terms of elevation. The zone B is mountainous and more than 35% of this area has a topographic slope above 25 degrees, but the slope above 25 degrees only covers 15% of zone A (Figure 1). This might explain the reason why the interval intensity in zone A was much greater than that in zone B during the second and third periods. In addition, the woodland is mainly found in elevated areas, and agricultural activities are normally found in flat area. To some extent, this can explain the reason why transition from cropland to built-up was systematic, but the transition from woodland to built-up was systematic avoided in all cases.

The surrounding land use type is the most frequently considered factor. Previous studies had reported that places are more likely to be developed when they are surrounded by urban land area [31,32,33]. This finding is consistent with our study result. As shown in Figure 2, the urban land in both zones was surrounded by the cropland and woodland. However, the woodlands are mainly located in high elevated areas in both zones. The urban expansion tends to take place at the expense of the cropland. The urban expansion in zone A tends to take place on the water and mudflat with lower costs. This can explain the reason why the transitions from water to built-up and from mudflat to built-up were targeted and the transition intensities increase gradually. This indicates that the high intensity of reclamation took place in zone A for all time periods.

**Figure 9:**
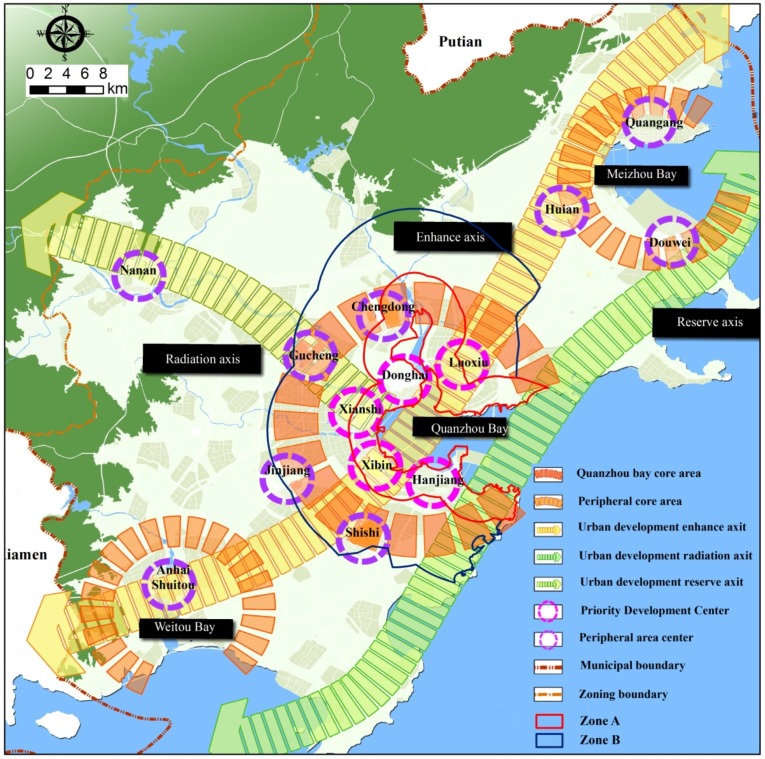
Urban development map (modified from “The master plan of Quanzhou city (2008–2030)”).

Urban planning and land use policy are important human-drivers for land change [34,35]. In 1989, 1995, and 2008, the Quanzhou government developed three master plans for Quanzhou city. According to the master plan developed in 1989, urban sprawling was designed to use the area surrounding Licheng and Fengzhe districts which were included as some part of zone A and zone B of this study. The results of interval level intensity for the first period 1990–2002 are in agreement with the design of this master plan. According to the master plan developed in 1995 and in 2008, the government issued a priority to develop Quanzhou bay surrounding areas shown on Figure 9 which included some part of zone A. This can explain why land use change in zone A is more intensive than zone B during the second and third intervals.

It is known that land use policy drives land change. China initiated the “Grain for Green Project” in 1999 to reduce soil erosion and increase forest coverage by removing lands with steep slopes and marginal farmlands from agricultural production [36]. After one year of implementing of this project, some land transformation started to happen showing reduction of cropland, partial conversion of cultivated land into woodland and grassland (Figure 6). The related requirement associated with the Protection of Basic Farmland policy was initiated in 1999 and demarcation the boundaries of “basic farmland protection area” to prevent the removal of cropland. It is regrettable to find that the total area of cropland still experienced a sharp decrease for all time periods in both zones around Quanzhou Bay in the context of urbanization (Table 3 and Table 4; Figure 7).

Before the 1980s, the naturally grown mangroves were less disturbed by human activities. Affected by the development of mudflat aquaculture, a large area of mangrove had been destroyed during the late 1980s. More recently, the local government has paid considerable attention to the ecological health and has initiated some conservative measures to protect mangroves ecosystem, e.g., the establishment of the “wetland natural protection area” at Luoyang river estuary in 1998 and the establishment of “estuary wetland provincial nature reserve area” at Quanzhou Bay in 2003 [37]. With the support of Fujian province government, the area of mangrove expanded from an initial area of 0.01% in 1990 to a total area of 1.1% in 2017. This supports the finding of this study that mangrove tends to increase in Quanzhou bay (Table 3 and Table 4).

Ocean-related policy definitely influences land development in coastal zone. “The Law of the People’s Republic of China on the Administration of Sea Areas” promulgated in 2002 is of great significance to the national construction of marine legal system. Since the implementation of this law, the marine economy and marine environment have changed greatly, as a result of nationwide sea reclamation (demonstrated as a case in coastal zone around Quanzhou Bay in this study) at the expense of aquaculture and mudflats (Figure 5). The total amount of sea area use fees was increased from 120 million in 2002 to 8206 million in 2015 [38]. This can explain why aquaculture, mudflat, and water experienced sharp decrease during the second and third time intervals. Meanwhile, this is consistent with the result that infrastructural gain targeted aquaculture except in the first time interval (1990–2002).

### 4.3. Management Implications

Based on the outcome resulted from this study, policy makers need to find a balanced point to solve the conflict interest between economic development and ecological conservation, and they also need to consider the socio-demographic structure and natural restrictions. Woodland, cropland, mudflat, and water have decreased while built-up has increased in both zones. This suggests that urban land tended to replace these land area rapidly in Quanzhou Bay areas. Mangroves have increased during all three time intervals, indicating that mangrove protection efforts are effective.

Both zones experienced substantial sharply gross losses to cropland. It is known that the dynamic modifications of cropland and the level of land-use intensity are the key factors that can influence sustainable development and food security [39], which has become a focus of the Chinese government. Knowing that cropland shrunk in two zones, reclamation and urbanization should consider the costs and benefits to farmers and rural residents in Quanzhou coastal bay area, especially for the transition from cropland to built-up.

Additional work should be initiated to enhance coastal ecosystem services. At both national and regional levels, establishing levels of concern regulated by the Ministry of Environmental Protection could reasonably balance economic development and ecological system conservation. It is the center of gravity for ecological civilization in China. Land is the spatial foundation for ecological civilization construction. Under the guidance of ecological civilization strategy, effective implementation of development priority zones and optimal land use layout are needed. Meanwhile, there is an urgent need to strengthen ecological protection and restoration, e.g., establishing estuarine wetland ecological protected area and regulating anthropogenic disturbances to avoid irreversible damage to the coastal ecosystem as a result of urban sprawl, thereby keeping a sound balance between natural ecosystems and human activities. Thus, a wise land use policy for sustainable urban development should be proposed in Quanzhou. This investigation provides implications for future research into the patterns and driving factors of LUCC in other regions that face similar situations.

## 5. Conclusions

Analysis with the method of land use dynamic degree indices can enhance a quantitative understanding on land-use change degree and urban expansion rate, but it fails to reveal systematic processes of land changes. Intensity analysis can reveal underlying processes within land changes. Results from both land use dynamic degree indices and intensity analysis show that land change was accelerating for all time periods in zone A, while land change was fastest for the second interval in zone B. Woodland and cropland experienced a net decrease for all time intervals in both two zones. The transition from cropland to built-up was systematically targeted and stationary while the transition from woodland to built-up was systematically avoiding transition in both zones. Built-up tended to target aquaculture for the second and third time intervals in zone B but avoid aquaculture for all intervals in zone A. It should be noted that although built-up tended to avoid water, the intensity of water’s loss increased for all time intervals in zone A. This may indicate gradual increase of reclamation in Quanzhou bay area. In general, land change in zone A was more significant than that in zone B during the second and third time intervals. Mangroves had been protected and had grown since a series of protection measures were implemented inside coastal zone.

Apparently, the potential ecological consequences of land use due to the spatial expansion of cities should be expected. If Quanzhou wants to continuously grow its economy and to protect the environment, attention should be paid to the loss of cropland and mudflat and degradation of the woodland and the loss of valuable ecosystem services. Based on the outcome resulted from this study, policy makers need to find the solutions to the conflicts between economic development and ecological conservation. The dynamics of agriculture and aquaculture must be managed carefully, and the farmers’ benefits should be considered before urbanization occurs. This investigation could provide implications for sustainable land management programs in similar coastal areas.

## Figures and Tables

**Table 1:** Landsat satellite imagery.

Date	(Path,Row)	Landsat Source	Spatial Resolution (m)
1990/12/25	(120,43)	5 TM	17.3
2002/11/05	(120,43)	5 TM	17.3
2009/07/20	(120,43)	7 ETM+	4.32
2017/02/31	(120,43)	7 ETM+	4.32

**Table 2:** Three indices to measure land use change in this study.

Indices	Equations	Notation
Single land-use dynamic degree (K)	K = $\frac{U_{b}-U_{a}}{U_{a}}*\frac{1}{T}*100\%$	T = time period; U_a_ = the area of this land use at the beginning of this period; U_b_ = the area of this land use at the end of this period; K represents the annual rate of change for this land use type in the study area.
Comprehensive land-use dynamic degree (S)	$S\;=\;\left\{\overset{n}{\underset{\mathrm{ij}}{\sum }}\left(\frac{\Delta S_{i-j}}{S_{i}}\right)\right\}*100*\frac{1}{T}*100\%$	S = comprehensive land-use dynamic degree for this time interval; S_i_ = the area of the *i*th land use type at the beginning of the monitoring period; n = number of categories; ΔS_i−j_ = total area of land type *i*converted into other types for this time period.
Annual intensity of urban expansion (SI)	$\mathrm{SI}\;=\;\frac{C_{\left(t+1\right)}-C_{t}}{C_{t}}*\frac{1}{T}*100\%$	SI = annual intensity of urban expansion; C_(t+1)_ and C_t_ are the area of built-up at time point t and t+1, respectively; T = time period.

**Table 3:** Land uses in 1990, 2002, 2009, and 2017 of Quanzhou bay coastal zone (%).

Land Use Type	1990		2002		2009		2017	
	Zone A	Zone B	Zone A	Zone B	Zone A	Zone B	Zone A	Zone B
Woodland	24.4	38.6	24.1	37.1	21.3	34.1	19.4	31.8
Cropland	29.1	20.3	25.3	17.0	16.3	12.0	11.8	9.6
Water	3.3	2.4	3.1	2.4	3.1	2.3	2.8	2.2
Built-up	29.2	38.1	33.9	42.9	48.9	51.1	56.3	56.0
Aquaculture	5.9	0.3	5.8	0.3	4.9	0.3	5.9	0.2
Mudflat	8.0	0.4	7.7	0.3	5.1	0.3	2.5	0.3
Mangroves	0.01		0.03		0.5		1.1	

**Table 4:** Results from three indices to measure land-use dynamic degree and urban expansion intensity.

Land-Use Category	Single Land-Use Dynamic Degree (K, %)					
	1990–2002		2002–2009		2009–2017	
	Zone A	Zone B	Zone A	Zone B	Zone A	Zone B
Woodland	−0.10	−0.33	−1.69	−1.16	−1.08	−0.84
Cropland	−1.10	−1.35	−5.06	−4.19	−3.45	−2.54
Water	−0.48	−0.06	−0.24	−0.69	−1.05	−0.34
Built-up	1.34	1.07	6.30	2.72	1.91	1.20
Aquaculture	−0.09	0.00	−2.36	−2.39	2.75	−4.27
Mudflat	−0.33	−0.59	−4.74	−0.81	−6.32	0.00
Mangrove	13.05		224.28		18.05	
Annual intensity of urban expansion (SI, %)	1.34	1.07	6.30	2.72	1.91	1.20
Comprehensive Land-use dynamic degree (S, %)	2.15	2.34	13.01	9.24	15.74	8.08
